# High Rates of Tuberculosis in End-Stage Renal Failure: the Impact of International Migration

**DOI:** 10.3201/eid0801.010017

**Published:** 2002-01

**Authors:** David A.J. Moore, Liz Lightstone, Babak Javid, Jon S. Friedland

**Affiliations:** Imperial College of Science, Technology and Medicine, Hammersmith Hospital, London, United Kingdom

**Keywords:** tuberculosis, renal failure, renal dialysis, immigrants, nosocomial

## Abstract

We studied a cohort of patients requiring renal dialysis who had migrated to the United Kingdom from tuberculosis (TB)-endemic countries and found extremely high rates of TB (1,187 cases per 100,000 per year), partly associated with end-stage diabetic renal disease. We recommend enhanced vigilance and screening of such patients, both to reduce illness and death and to prevent nosocomial spread of TB among susceptible persons.

Immigrants, asylum seekers, and refugees from war and oppression often originate in countries where tuberculosis (TB) is endemic and relocate to more affluent countries, where the incidence of TB is low. A proportion of such persons will have or develop renal failure requiring renal replacement therapy (hemodialysis or continuous ambulatory peritoneal dialysis [CAPD]). Chronic renal failure impairs immune function and is associated with an increased incidence of TB. Among patients with chronic renal failure requiring renal replacement therapy, rates of TB 10- to 25-fold greater than those in the general population have been reported from the United States, Canada, Europe, and Japan, equating to incidence rates of approximately 250 cases per 100,000 per year (*1*–*3*). In areas of TB endemicity, the relative risk attributable to chronic renal failure requiring dialysis appears to be similar (*4*), although as the background rate in these settings is so much greater the absolute rate will be considerably higher. The impact of international migration and the increase in asylum seekers and refugees on the incidence of TB in renal dialysis patients has not been previously explored.

##  The Study

We investigated the incidence of TB in patients referred for dialysis over a 5-year period (April 1, 1994, to March 31, 1999) to the Hammersmith Hospital, situated in an area of London that is home to many immigrants, refugees, and asylum seekers. A mean of 156 patients underwent dialysis in the Hammersmith Dialysis Unit each year of the study; a mean of 45 patients were new to the program each year. Patients in the renal replacement therapy cohort database who had been diagnosed with and treated for TB were identified by manual searching of hospital microbiology and histopathology databases and the handwritten logbook in which all local reports to the U.K. Communicable Disease Surveillance Centre are recorded. Duration of follow-up under the care of the renal unit (within the specified 5-year study period) was calculated for each patient. The length of time that patients had been resident in the United Kingdom was not available. Eleven TB cases (two fatal; the remainder making a complete recovery following treatment) were identified from 431 dialysis patients, including 191 who received peritoneal dialysis (CAPD). The incidence of TB was 1,187 cases per 100,000 renal patients per year. Case notes for all but one patient diagnosed with TB were available for review.

Patients had been on dialysis for 24.5 ±8.5 months (± standard error of the mean). During the study period, the total dialysis population at the Hammersmith Hospital comprised 41% Caucasians, 40% Asians, 18% black Caribbeans, and 1% from other parts of the world, including six from the Indian subcontinent, three from sub-Saharan Africa, and one from the Republic of Ireland. All the TB patients were born overseas. During the period of study, patients were not consistently screened for TB at time of entry into the United Kingdom. Specific screening of renal failure patients was also not routine, and no patient in our group received any form of chemoprophylaxis. In general, purified protein derivative (PPD) testing is not routine in the United Kingdom, where most of the population has had BCG vaccination or exposure to infection. PPD responses are less useful in the renal failure population, who tend to be anergic. Two of our patients did have PPD tests during diagnostic workup; results were negative.

Patients were treated with standard quadruple therapy of isoniazid, rifampicin, pyrazinamide, and ethambutol for 2 months; isoniazid and rifampicin treatment continued for 4 to 10 months thereafter. Once a fully sensitive pathogen had been cultured, ethambutol was discontinued. Nine of the 11 TB patients had fully drug-sensitive organisms. For the other two, diagnosis was histologic only, but complete cure followed standard therapy. However, there were some delays in diagnosis, and one patient died before the diagnosis was confirmed by culture of *Mycobacterium tuberculosis* from peritoneal fluid. Despite this, infection did not develop in any staff or other patient contacts found by contact tracing. In four (40%) of the TB patients, the underlying cause of renal failure was diabetes mellitus, although only 19% of the total dialysis population had diabetes (13% of Caucasians, 24% Asians, and 19% Afro-Caribbeans). Other causes of renal failure included hypertension, chronic pyelonephritis, immunoglobulin A nephropathy, and HIV nephropathy (only one of six patients tested was HIV seropositive). When first seen, six patients had pulmonary or pleural TB, including one patient with empyema. Four patients had peritoneal, two lymph node, and one hepatic TB. Of the six patients with pulmonary TB, one also had peritoneal TB and one had concomitant lymph-node infection. No evidence suggested an outbreak of TB, and no clustering of cases occurred (three cases occurred in 1994, two in 1995, one in 1996, three in 1997, and two in 1998).

## Conclusions

Our study showed that the incidence of TB in patients requiring dialysis is extremely high and 100-fold greater than the incidence of TB in the general population of England and Wales, which was 12 per 100,000 in 1998 (*5*). These figures are probably an underestimate of the extent of TB in renal disease since ascertainment was based on definitive microbiologic or histologic confirmation of diagnosis and legally required reports. We cannot exclude the possibility that occasional patients were treated on the basis of clinical suspicion and not reported. Nor did we evaluate TB cases in dialysis-independent patients with renal failure.

Part of the explanation for our findings is that patients came from countries where TB is endemic. However, TB incidence rates in England and Wales from 1988 to 1998 were, at the most, 210 and 132 per 100,000 population among black African patients and patients from the Indian subcontinent, respectively (*5*), figures which are substantially less than in our renal replacement therapy patients. Two other factors likely to contribute to the high rates of TB are renal failure (*1*–*3*) and the high prevalence of diabetes mellitus in patients from the Indian subcontinent, a group in whom we have noted a high incidence of end-stage renal disease (*6*). Diabetes mellitus appears to be associated with TB in patients with renal failure (*7*). Although most disease was pulmonary, peritoneal TB in CAPD patients is relatively common, and the diagnosis should always be considered in patients with persistent conventional culture-negative peritonitis.

Two of our patients died, which may reflect the fact that the clinical symptoms of TB can be difficult to distinguish from those of uremia, causing delay in diagnosis, although dialysis-dependent renal failure has been shown to be a potent contributor to death (*8*). HIV infection is an infrequent cause of chronic renal failure among patients at the Hammersmith Hospital and was not a major influence on TB reactivation in this patient group. However, our data suggest that renal failure requiring renal replacement therapy is as potent a risk factor for reactivation of TB as any (including HIV infection) previously described.

There are two important messages from this study, which showed that renal failure in immigrants is associated with extremely high rates of TB in the context of migration from disease-endemic areas and a high incidence of diabetes mellitus. First, it is critical that screening for latent TB infection in patients with renal failure occur at a very early stage in people who come from areas of the world where TB is endemic, a recommendation in line with those of the American Thoracic Society, Centers for Disease Control and Prevention, and the Council of the Infectious Diseases Society of America (*9*). Treatment for latent TB infection should be routinely considered in such patients. Although the sensitivity of TB skin testing is substantially reduced in the setting of chronic renal failure with rates of anergy in excess of 30% (*10*), this should not preclude its use as a screening tool because specificity is unaltered. Second, since chronic renal failure necessitates frequent attendance at medical facilities, the potential for nosocomial spread among susceptible persons is considerable. Enhanced vigilance is necessary to ensure early diagnosis of infectious cases and prompt institution of appropriate therapy.
